# Supplementary material for: Modeling glioblastoma heterogeneity as a dynamic network of cell states
Source: Mol Syst Biol. 2021 Sep 16;17(9):e10105. doi: 10.15252/msb.202010105 (PMC8444284; doi:10.15252/msb.202010105)
Supplement: Supplementary file 5 — Source Data for Figure 3 [file MSB-17-e10105-s001.zip › Figure3A_sourcedata/GSEA_3065/nosigdb_state3.GseaPreranked.1622718426028/OLSEN_MESENCHYMAL.html]

Details for gene set OLSEN\_MESENCHYMAL[GSEA]

|  || Dataset | state3 |
| Phenotype | NoPhenotypeAvailable |
| Upregulated in class | na\_neg |
| GeneSet | OLSEN\_MESENCHYMAL |
| Enrichment Score (ES) | -0.48176342 |
| Normalized Enrichment Score (NES) | -1.6252474 |
| Nominal p-value | 0.004651163 |
| FDR q-value | 0.026360787 |
| FWER p-Value | 0.39 |
Table: GSEA Results Summary

  

Fig 1: Enrichment plot: OLSEN\_MESENCHYMAL      
 Profile of the Running ES Score & Positions of GeneSet Members on the Rank Ordered List

  

| PROBE | GENE SYMBOL | GENE\_TITLE | RANK IN GENE LIST | RANK METRIC SCORE | RUNNING ES | CORE ENRICHMENT || 1 | B2M |  |  | 11 | 0.538 | 0.0308 | No |
| 2 | POSTN |  |  | 24 | 0.487 | 0.0586 | No |
| 3 | CD63 |  |  | 26 | 0.480 | 0.0871 | No |
| 4 | TIMP1 |  |  | 35 | 0.470 | 0.1142 | No |
| 5 | ITM2B |  |  | 82 | 0.361 | 0.1304 | No |
| 6 | CALD1 |  |  | 124 | 0.322 | 0.1449 | No |
| 7 | RRBP1 |  |  | 220 | 0.274 | 0.1503 | No |
| 8 | COL4A2 |  |  | 232 | 0.271 | 0.1652 | No |
| 9 | COL4A1 |  |  | 241 | 0.267 | 0.1802 | No |
| 10 | COL6A1 |  |  | 254 | 0.261 | 0.1944 | No |
| 11 | COL5A2 |  |  | 266 | 0.255 | 0.2083 | No |
| 12 | VCAN |  |  | 293 | 0.245 | 0.2200 | No |
| 13 | COL5A1 |  |  | 302 | 0.242 | 0.2335 | No |
| 14 | THY1 |  |  | 326 | 0.236 | 0.2449 | No |
| 15 | SPARC |  |  | 361 | 0.228 | 0.2546 | No |
| 16 | PCOLCE |  |  | 533 | 0.194 | 0.2465 | No |
| 17 | SERPINH1 |  |  | 588 | 0.188 | 0.2515 | No |
| 18 | LIMA1 |  |  | 639 | 0.180 | 0.2565 | No |
| 19 | CST3 |  |  | 749 | 0.164 | 0.2538 | No |
| 20 | FSTL1 |  |  | 790 | 0.159 | 0.2586 | No |
| 21 | NEAT1 |  |  | 855 | 0.152 | 0.2603 | No |
| 22 | TIMP3 |  |  | 950 | 0.142 | 0.2580 | No |
| 23 | CCDC80 |  |  | 966 | 0.141 | 0.2647 | No |
| 24 | FN1 |  |  | 973 | 0.140 | 0.2724 | No |
| 25 | EMP1 |  |  | 1624 | 0.096 | 0.2033 | No |
| 26 | NPC2 |  |  | 1842 | 0.084 | 0.1833 | No |
| 27 | PRSS23 |  |  | 1890 | 0.081 | 0.1827 | No |
| 28 | COL14A1 |  |  | 2159 | 0.070 | 0.1560 | No |
| 29 | PPIC |  |  | 2162 | 0.070 | 0.1600 | No |
| 30 | AEBP1 |  |  | 2257 | 0.066 | 0.1531 | No |
| 31 | C1R |  |  | 2407 | 0.060 | 0.1395 | No |
| 32 | COL6A2 |  |  | 2565 | 0.055 | 0.1247 | No |
| 33 | UACA |  |  | 2971 | 0.042 | 0.0806 | No |
| 34 | IFITM3 |  |  | 3449 | 0.029 | 0.0274 | No |
| 35 | IGFBP5 |  |  | 3555 | 0.027 | 0.0169 | No |
| 36 | NNMT |  |  | 3707 | 0.022 | 0.0008 | No |
| 37 | FOS |  |  | 3899 | 0.018 | -0.0201 | No |
| 38 | GSN |  |  | 4064 | 0.013 | -0.0382 | No |
| 39 | EGR1 |  |  | 4134 | 0.012 | -0.0455 | No |
| 40 | JUNB |  |  | 4467 | 0.002 | -0.0836 | No |
| 41 | LY6E |  |  | 4707 | -0.004 | -0.1109 | No |
| 42 | IFITM2 |  |  | 5106 | -0.014 | -0.1559 | No |
| 43 | IGFBP7 |  |  | 5667 | -0.029 | -0.2186 | No |
| 44 | SELENOM |  |  | 5731 | -0.031 | -0.2241 | No |
| 45 | TPM2 |  |  | 5823 | -0.034 | -0.2325 | No |
| 46 | LHFPL6 |  |  | 5831 | -0.034 | -0.2313 | No |
| 47 | GADD45B |  |  | 6619 | -0.062 | -0.3183 | No |
| 48 | TAGLN |  |  | 6722 | -0.067 | -0.3260 | No |
| 49 | ZFP36L1 |  |  | 6893 | -0.076 | -0.3411 | No |
| 50 | ANXA1 |  |  | 7032 | -0.083 | -0.3520 | No |
| 51 | LMNA |  |  | 7412 | -0.109 | -0.3891 | No |
| 52 | PRRX1 |  |  | 7670 | -0.136 | -0.4107 | No |
| 53 | EEF1D |  |  | 7817 | -0.154 | -0.4183 | No |
| 54 | CAVIN1 |  |  | 8208 | -0.231 | -0.4494 | No |
| 55 | S100A11 |  |  | 8490 | -0.336 | -0.4617 | Yes |
| 56 | ID3 |  |  | 8502 | -0.341 | -0.4426 | Yes |
| 57 | MT2A |  |  | 8650 | -0.456 | -0.4324 | Yes |
| 58 | S100A13 |  |  | 8652 | -0.457 | -0.4052 | Yes |
| 59 | RPS25 |  |  | 8661 | -0.467 | -0.3783 | Yes |
| 60 | RPL32 |  |  | 8715 | -0.556 | -0.3512 | Yes |
| 61 | SERF2 |  |  | 8724 | -0.594 | -0.3167 | Yes |
| 62 | RPLP1 |  |  | 8728 | -0.606 | -0.2809 | Yes |
| 63 | S100A6 |  |  | 8729 | -0.615 | -0.2442 | Yes |
| 64 | TPT1 |  |  | 8735 | -0.644 | -0.2063 | Yes |
| 65 | RPL41 |  |  | 8743 | -0.718 | -0.1643 | Yes |
| 66 | LGALS1 |  |  | 8744 | -0.718 | -0.1214 | Yes |
| 67 | TMSB4X |  |  | 8748 | -0.920 | -0.0669 | Yes |
| 68 | FTL |  |  | 8751 | -1.124 | 0.0000 | Yes |
Table: GSEA details [plain text format]

  

Fig 2: OLSEN\_MESENCHYMAL: Random ES distribution      
 Gene set null distribution of ES for **OLSEN\_MESENCHYMAL**
